# Case Report: The price of intraoperative cell salvage? - implantation metastases of meningioma in both lungs and left cubital fossa

**DOI:** 10.3389/fonc.2025.1535048

**Published:** 2025-05-28

**Authors:** Mengyao He, Shengping Yu, Wenjun Luo, Yu Zhang, Wenchao Liu, Cuiyun Sun, Qiang Huang

**Affiliations:** ^1^ Department of Neurosurgery, Tianjin Medical University General Hospital, Tianjin, China; ^2^ Department of Neuropathology, Tianjin Medical University General Hospital, Tianjin, China

**Keywords:** extracranial dissemination, intraoperative cell salvage, meningioma, metastasis, TERT promoter mutation

## Abstract

Meningiomas are common Central Nervous System (CNS) tumors, typically benign, but they can occasionally metastasize or exhibit aggressive behavior. We present a case of meningioma with metastasis to unique sites (pulmonary and left cubital fossa) and intracranial recurrent progression. This case highlights challenges in managing recurrent and metastatic meningioma and the potential risks of intraoperative cell salvage (IOCS).

## 1 Introduction

Meningiomas are the most common benign CNS tumors, accounting for 40.8% of all tumors and 56.2% of benign tumor (1). The World Health Organization (WHO) 2021 classification emphasizes molecular diagnostics, reshaping diagnostic criteria (2, 3). Deletions of cyclin-dependent kinase inhibitor (*CDKN2A/B*) indicate aggressive behavior (4), while telomerase reverse transcriptase (*TERT*) promoter mutations are markers of high recurrence risk. Meningiomas with these mutations are classified as WHO Grade 3 (5–7). Despite their generally benign nature, meningiomas can recur and metastasize, with less than 1% of cases exhibiting distant spread, typically to the lungs, liver, lymph nodes, and bones (8–11). The likelihood of metastatic spread in meningiomas is correlated with factors such as WHO grading (12). This report presents a case of bilateral pulmonary and left cubital fossa metastases following meningioma surgery, along with intracranial recurrence and upgrade in grade, summarized as follows.

## 2 Case description

**Figure 1:**
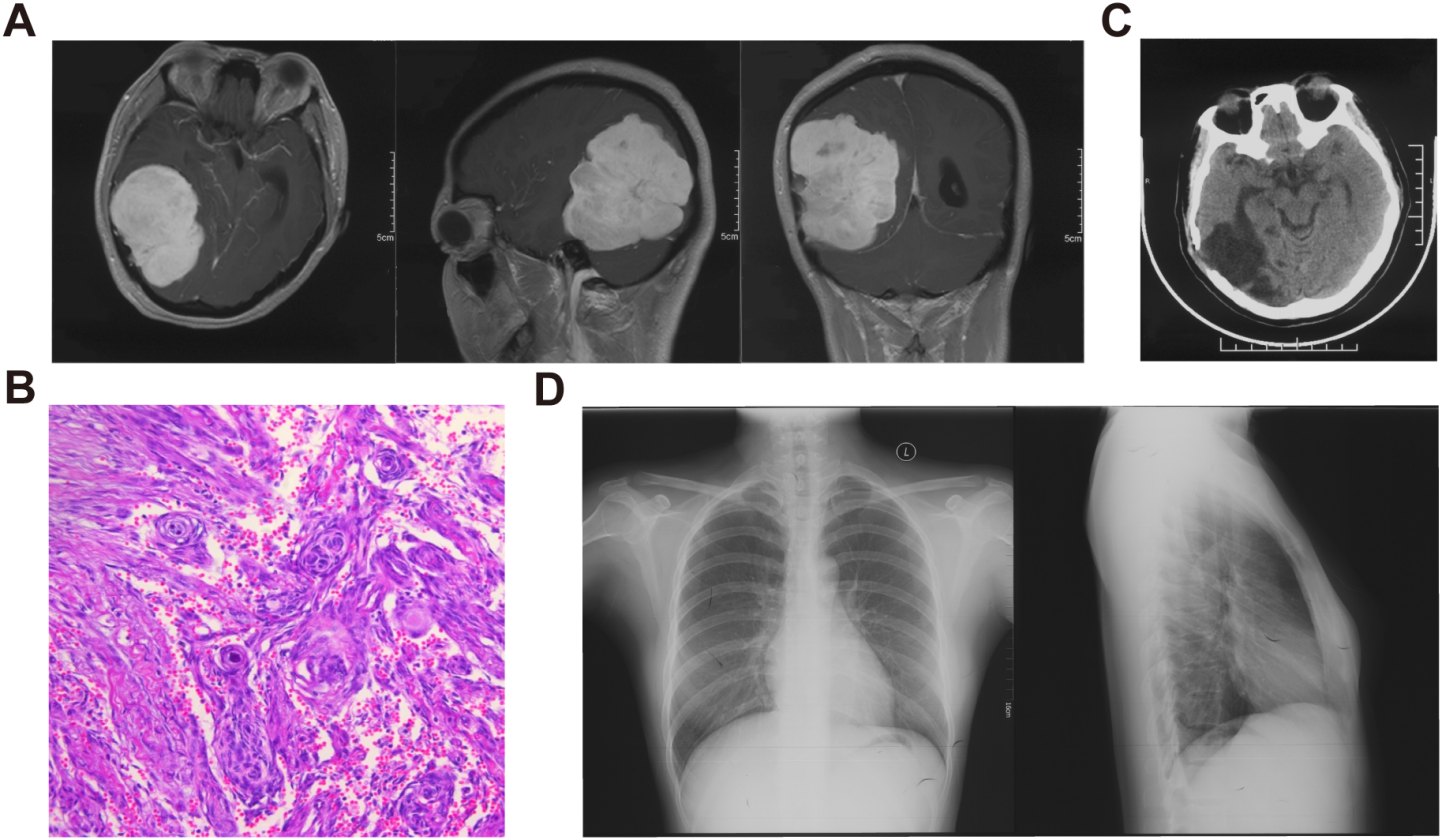
Imaging and pathological data of the patient’s initial meningioma. **(A)** Preoperative enhanced MRI of the head showing a large intracranial mass with clear borders and mixed density within the lesion. **(B)** HE staining revealing a typical meningioma whirl, confirming the diagnosis of fibrous meningioma. **(C)** One-year post-operative follow-up head CT. **(D)** Chest X-ray showing no significant abnormalities.

The patient had no long-term exposure to exogenous progesterone had no family history of meningioma. Eight years ago, she underwent resection of a large mass in the right parieto-occipital region (**Figure 1A**). The tumor was resected in fragments, resulting in significant blood loss. The surgical team collected and processed the blood loss from the operative field for reinfusion. Pathology confirmed a WHO Grade 1 fibrous meningioma (**Figure 1B**), and no adjuvant radiotherapy (RT) was given. At a one-year postoperative follow-up, a head CT revealed no significant signs of recurrence (**Figure 1C**), and a concurrent chest X-ray showed no significant abnormalities (**Figure 1D**).

**Figure 2:**
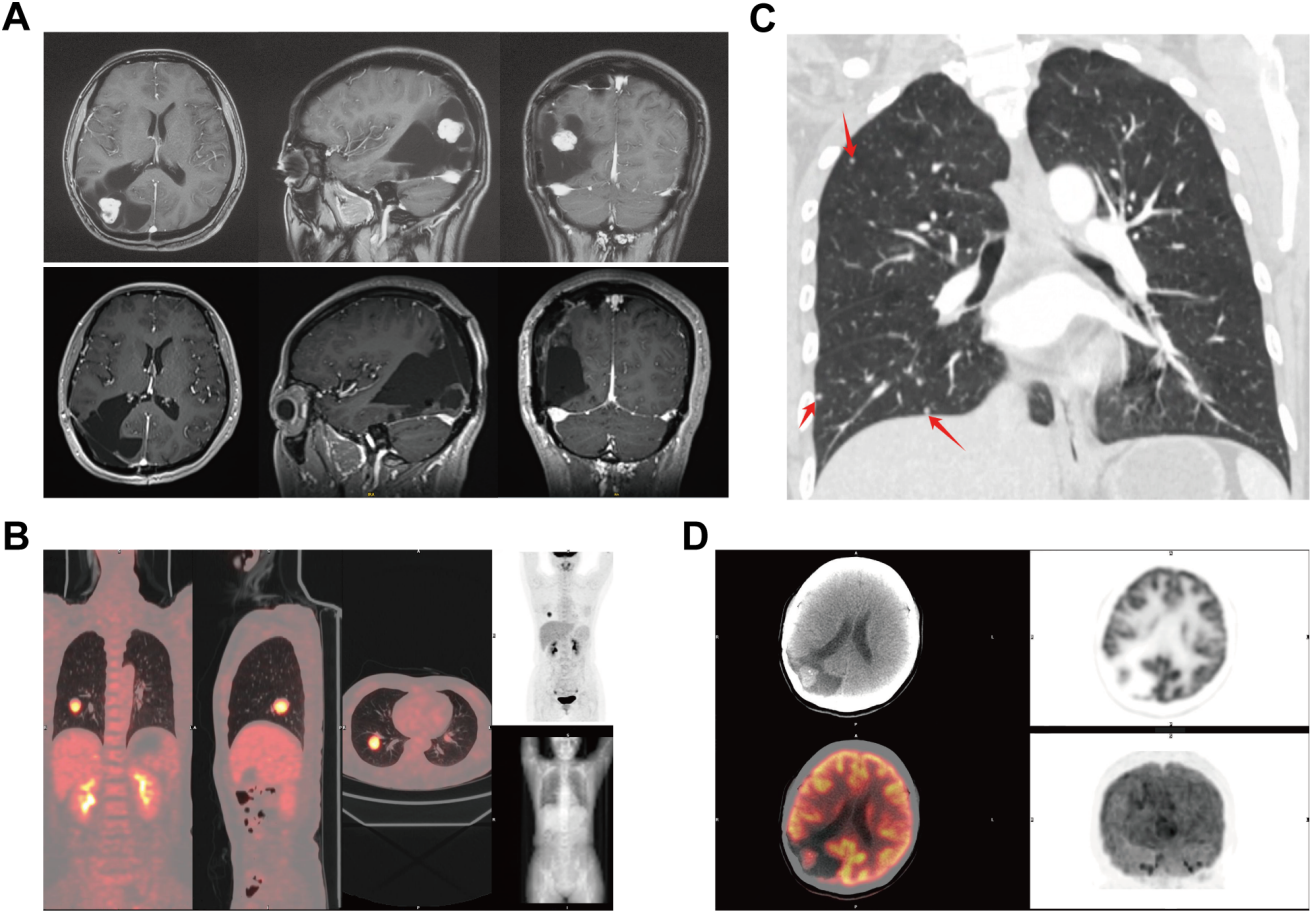
Imaging of recurrent meningioma in the head and body. **(A)** Pre - and post-operative enhanced MRI of the head. **(B)** The largest density nodule shows localized increased metabolism. **(C)** Multiple nodular shadows in both lungs (indicated by red arrows) distributed in the peripheral lung zones. **(D)** PET-CT showing increased metabolic activity in the right parieto-occipital lesion.

Recently, she experienced dizziness and nausea. MRI suggested possible meningioma recurrence (**Figure 2A**). Systematic physical examination revealed a round mass in the left cubital fossa, first noted three years prior. CT showed multiple solid nodules in both lungs, the largest with a maximum diameter of 30 mm in the right lower lobe (**Figures 2B, C**). Positron emission tomography-computed tomography (PET-CT) indicated increased metabolic activity in both intracranial and pulmonary lesions (**Figures 2B, D**), with a maximum standardized uptake value (SUV) of 7.9, strongly suggesting the possibility of pulmonary metastasis from the meningioma.

**Figure 3:**
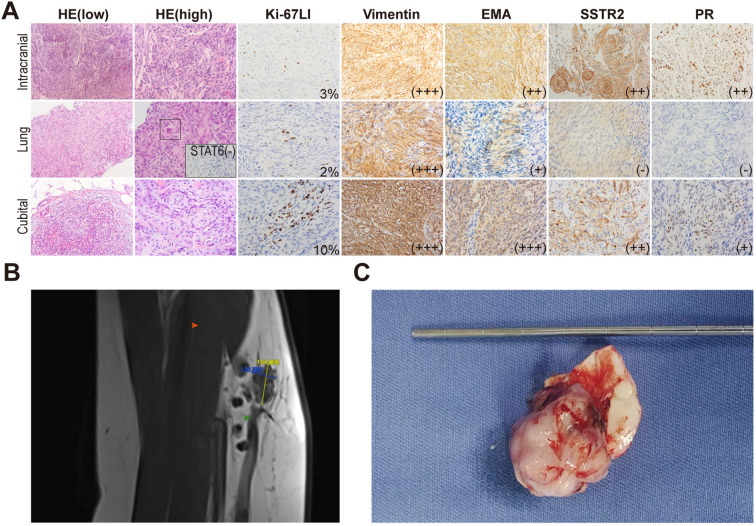
Characteristics and comparison of three lesions. **(A)** HE and immunohistochemical staining. In the pulmonary lesion, an atypical meningeal whirlpool was observed under high-power HE staining (shown in the black box). STAT6 was performed to exclude the possibility of solitary fibrous tumor. **(B)** An irregular nodular shadow on the anterior medial side of the left cubital fossa, with scattered nodular shadows of varying sizes. Red arrow points the biceps brachii. Green arrow points the brachial vein. **(C)** Gross specimen of the intracranial lesion, showing a well-formed capsule connected to the meninges.

Lung lesions and left cubital fossa mass confirmed meningioma metastases (**Figures 3A, B**). A Simpson I resection of the intracranial lesion was performed, confirming atypical meningioma (**Figures 3A, C**). Next-generation sequencing (NGS) revealed a c.-146C>T mutation in the *TERT* promoter, indicating aggressive behavior. The mutation rates of the *TERT* promoter and *CDKN*2A/B in meningiomas are typically less than 5% and are often co-occurring with Neurofibromin 2 (*NF2*) mutations and/or 22q chromosome deletions. But the karyotype analysis of this patient showed no significant abnormalities. The final diagnosis was CNS 5^th^ WHO Grade 3 anaplastic meningioma with multiple metastases.

Postoperatively, the patient’s head was treated with a conventional RT regimen of 2 Gray (Gy) for 28 sessions. Five metastatic lesions in the lower lobe of the left lung received stereotactic body radiation therapy (SBRT) at a dose of 60Gy in 8 fractions. Additionally, two lesions in the upper lobe of the left lung each received one session of interstitial brachytherapy (ISBT), aiming at controlling the total radiation dose to the lungs. The subsequent treatment plan involves continuing the ISBT for the lung and completing the RT to the left cubital fossa region, with a scheduled resection of the largest pulmonary lesion in the right lower lung to obtain primary tumor cells for drug sensitivity testing, aiming to identify highly sensitive systemic therapies. The patient is highly cooperative with the current treatment. As of November 2024, the patient maintained a Karnofsky Performance Score of 90, with no evidence of radiation pneumonitis or other acute toxicities of grade ≥2. Pulmonary function tests demonstrated stable FEV1/FVC ratios, consistent with baseline values. Serial imaging surveillance revealed no significant progression of the previously documented lesions.

## 3 Discussion

This report describes a case of meningioma with metastasis to unique sites (pulmonary and left cubital fossa) and intracranial recurrent progression. Despite no history of prolonged exogenous progesterone exposure, genetic testing revealed a deletion of the *NF2*, indicating it may be a high-risk factor for this meningioma. A meta-analysis indicated that median recurrence-free survival for patients with *TERT* mutations is 14 months, compared to 101 months for those with wild-type *TERT*. While some cohort studies suggest that *TERT* promoter mutations are typically not acquired during the process of malignant progression, they may occur independently of the malignant transformation of meningiomas (13). Generally, grade 1 meningiomas have a favorable prognosis after complete resection, and distant metastases are relatively rare (14). Nevertheless, considering the patient’s clinical history and disease progression, the likelihood of recurrence from a grade 1 meningioma advancing to grade 3 is significant. Additionally, it cannot be ruled out that the patient may have had *TERT* mutations at the initial presentation, which were not detected at that time. This situation highlighting the importance of comprehensive molecular testing. A multicenter study reported a 73% recurrence rate for grade 3 meningiomas within 46 months. However, postoperative RT was independently associated with improved overall survival (15), which emphasized personalized treatment plans. Additionally, the distinction between primary and secondary malignant meningiomas showed no significant overall survival difference (16), suggesting the need for improved diagnostic criteria integrating genetic markers for better therapeutic strategies.

In the most common metastatic site for meningiomas—the lungs—lesions rarely exceed 20 in number. Reports indicate favorable outcomes for surgical resections of lung metastases from meningiomas. However, due to the numerous small metastatic lesions in this patient, complete surgical eradication is challenging. Given the patient’s genetic testing and tumor grading, indiscriminate RT to the entire lung was deemed unfeasible, leading to the use of a combination of SBRT and localized ISBT to refine treatment while minimizing damage to surrounding tissues.

Additionally, the lesion in the patient’s left cubital fossa warrants attention. We conducted a systematic literature search through PubMed, Embase, and Web of Science using the keywords: meningioma + metastasis + cubital fossa/subcutaneous/elbow. No reports of meningioma metastases to distal subcutaneous sites, including the cubital fossa, have been previously published. Significant intraoperative bleeding during the first meningioma surgery, involving substantial transfusions and IOCS, may have facilitated tumor cell dissemination into the cubital fossa’s soft tissue spaces. We found that the ultrasound examination of the left upper limb vasculature conducted postoperatively revealed poorly defined vascular structures. This raises the possibility that the intraoperative reinfusion may have allowed tumor cells to disseminate into the soft tissue spaces of the cubital fossa via the bloodstream, subsequently leading to extensive spread in the lungs. The lung lesions were primarily located in the peripheral lung fields, consistent with the characteristics of hematogenous dissemination. While IOCS is widely used, for patients with CNS tumors, it carries a theoretical risk of promoting tumor cell dissemination. Studies indicate that viable tumor cells can persist even after leukocyte depletion filters (LDF) (17, 18). In this case, the patient underwent piecemeal resection during the first surgery without LDF, and no abnormal nodules or signs of recurrence were observed on the CT scan one year postoperatively, further supporting the possibility of intraoperative reinfusion leading to tumor dissemination. Therefore, proper preoperative preparation to minimize intraoperative bleeding and avoid large-volume transfusions is crucial for preventing tumor dissemination.

Regarding treatment, the intracranial lesions expressed somatostatin receptor 2, while lung metastases were negative, suggesting limited options for peptide receptor radionuclide therapy (19). The patient’s genetic profile predicts poor sensitivity to immunotherapy, yet insights from glioblastoma multiforme experiences may inform future strategies (20). Further genetic testing revealed a fibroblast growth factor receptor 3 amplification, which may respond to targeted therapy. The plan includes resecting the largest pulmonary lesion to obtain primary tumor cells for drug sensitivity testing and close monitoring post-treatment. If necessary, consideration may be given to experimental drug therapies, including sunitinib, bevacizumab, or multi-kinase inhibitors targeting vascular endothelial growth factor receptors.

Economic factors limited genetic testing of metastatic lesions, potentially overlooking critical information related to tumor heterogeneity or driver mutations. Future studies should employ multi-lesion molecular analysis to validate clonal origin and clarify the role of IOCS in metastasis.

## 4 Conclusion

This rare case of meningioma with bilateral pulmonary and left cubital fossa metastases, alongside intracranial recurrence and progression from grade 1 to grade 3, underscores the importance of comprehensive genetic analysis in diagnosis and treatment planning. Individualized RT and targeted systemic therapies should be prioritized to improve outcomes. This case also highlights the potential risks of tumor dissemination linked to IOCS in CNS tumor surgeries.

## Data Availability

The original contributions presented in the study are included in the article/supplementary material. Further inquiries can be directed to the corresponding author/s.
